# TcTI, a Kunitz-type trypsin inhibitor from cocoa associated with defense against pathogens

**DOI:** 10.1038/s41598-021-04700-y

**Published:** 2022-01-13

**Authors:** Milena do Amaral, Ana Camila Oliveira Freitas, Ariana Silva Santos, Everton Cruz dos Santos, Monaliza Macêdo Ferreira, Abelmon da Silva Gesteira, Karina Peres Gramacho, Jeanne Scardini Marinho-Prado, Carlos Priminho Pirovani

**Affiliations:** 1 Universidade Estadual de Santa Cruz, UESC, Rodovia Ilhéus-Itabuna, Km – 16, Ilhéus, BA CEP 45662-900 Brazil; 2 Instituto Nacional de Câncer José Alencar Gomes da Silva, Rio de Janeiro, RJ 20230-130 Brazil; 3 Embrapa Mandioca e Fruticultura, Cruz das Almas, BA 44380-000 Brazil; 4 Centro de Pesquisa do Cacau [CEPEC/CEPLAC] Molecular Plant Pathology Laboratory, Km 22 Rod. Ilhéus-Itabuna, Ilhéus, Bahia 45600-970 Brazil; 5 Embrapa Meio Ambiente, Jaguariúna, SP 13918-110 Brazil

**Keywords:** Biochemistry, Biotechnology, Computational biology and bioinformatics, Molecular biology, Plant sciences

## Abstract

Protease inhibitors (PIs) are important biotechnological tools of interest in agriculture. Usually they are the first proteins to be activated in plant-induced resistance against pathogens. Therefore, the aim of this study was to characterize a *Theobroma cacao* trypsin inhibitor called TcTI. The ORF has 740 bp encoding a protein with 219 amino acids, molecular weight of approximately 23 kDa. rTcTI was expressed in the soluble fraction of *Escherichia coli* strain *Rosetta *[*DE3*]*.* The purified His-Tag rTcTI showed inhibitory activity against commercial porcine trypsin. The kinetic model demonstrated that rTcTI is a competitive inhibitor, with a Ki value of 4.08 × 10^–7^ mol L^−1^. The thermostability analysis of rTcTI showed that 100% inhibitory activity was retained up to 60 °C and that at 70–80 °C, inhibitory activity remained above 50%. Circular dichroism analysis indicated that the protein is rich in loop structures and β-conformations. Furthermore, in vivo assays against *Helicoverpa armigera* larvae were also performed with rTcTI in 0.1 mg mL^−1^ spray solutions on leaf surfaces, which reduced larval growth by 70% compared to the control treatment. Trials with cocoa plants infected with *Mp* showed a greater accumulation of TcTI in resistant varieties of *T. cacao*, so this regulation may be associated with different isoforms of TcTI. This inhibitor has biochemical characteristics suitable for biotechnological applications as well as in resistance studies of *T. cacao* and other crops.

## Introduction

Protease inhibitors (PIs) are important targets with potential biotechnological use and application in agriculture^1,2^. Generally, they are the first proteins to be activated in plants as a defense mechanism against pathogens, promoting resistance. Trypsin inhibitors are associated with plants’ resistance to pathogens and are commonly known to act against insect pests by inhibiting proteases of the digestive tract, interfering in the pests’ development and proliferation^3–5^. PI trypsin interacts in canonical form with protease, forming a stable and reversible complex that confers high inhibitory capacity^6^.

This enzyme-inhibitor interaction participates in the regulation of multiple processes, such as plant-pathogen interactions, proteolysis of seed storage proteins and development and maturation of plant tissues^4,7,8^. The biochemical characterization of PI has been important for the understanding of these interaction processes. It is an important target for biotechnological use and application in agriculture^3^ Data from dissociation constant studies (Ki) indicate very low values of serine proteases inhibited by PIs. These values indicate that inhibition occurs satisfactorily, showing low dissociation of the enzyme-inhibitor complex^9^.

Serine protease inhibitors are grouped into 67 families. Those within the Kunitz family have been studied the most and are found in abundance in leguminous plants^10^. The inhibitors of the Kunitz family usually have 18–24 kDa, two disulfide linkages and low cysteine content^8^. These features are conserved among plant species, so these inhibitors have been widely studied for biotechnological applications^11,12^.

Serine protease inhibitors are involved in environmental responses, including during plant development^13^, and in plant defense responses against mites (*Tetranychus urticae*), as reported by Arnaiz et al.^14^, who observed that some types of KTIs identified in *Arabidopsis thaliana* were induced after infestation with *Tetranychus urticae*. Mechanical damage, insect attack and infection by microorganisms significantly increase the levels of PIs at the site of the attack in a systemic way^7,15^.

The PIs found in plants have the ability to inhibit endogenous enzymes, such as during seed development^16^. However, many plant inhibitors have specificity to exogenous proteases, since many of these inhibit herbivore proteases and pathogenic microorganisms^17–19^.

Much is known about the effect of trypsin inhibitors on plant defense against insects^4,7,20^, and many of them are also related to protection against fungal infestation^21–23^. Transgenic tobacco plants overexpressing a Kunitz-type trypsin inhibitor prevented the growth of the fungus *Rhizoctonia solani*^22^. Studies of wheat have demonstrated the inhibitory capacity of plant serine protease inhibitors (SPIs) on the growth of *Alternaria alternate* (which attacks tobacco plants) and *A. solani* (pathogenic to tomatoes). The different trypsin inhibitors investigated were able to interfere with spore germination and mycelial growth of tomato and tobacco fungi, respectively^24,25^. Other inhibitors belonging to the Kunitz-type family have been studied and shown inhibitory activity on growth and development of oomycetes of *Phytophthora infestans*, a pest that affects potato crops^1,26,27^.

Different classes of proteases and their inhibitor proteins are identified in genomic banks of cacao. In libraries of the EST interaction *Theobroma cacao* × *Moniliophthora perniciosa*^28,29^, sequences for trypsin inhibitors were only identified for the resistant variety of cacao. The Kunitz-type trypsin inhibitors were more abundant in the resistant cocoa genotype (TSH 1188) and none of these inhibitors were found in the susceptible genotype. Therefore, the trypsin inhibitors play a major role in the resistance of plants against the action of pathogens.

Studies conducted with cocoa seed extract, which is rich in protease inhibitors and accounts for 54% of the total proteins^30^, showed activity against the growth of larvae of Lepidopteran crop pests^31^. However, little is known about the specific function of cacao trypsin inhibitors (TI) in the control of pests and their interaction with the target serine protease. In contrast, cysteine protease and specific cystatin-like protease inhibitors have been characterized for the interaction between cacao and *Mp*, a causal agent of witches’ broom disease, such as protease-type legumain^32^, papain-like cysteine proteases and cysteine inhibitors^32,33^.

Due to the characteristics of the plant trypsin inhibitor, we propose that cocoa trypsin inhibitors should be associated with plant defense against the attack of pests and have promising characteristics for biotechnological applications. Therefore, this is the first study that biochemically characterizes a cocoa trypsin inhibitor (TcTI) by a heterologous system tracing its protein profile. The Kunitz-type trypsin inhibitor of *T. cacao* was cloned and expressed in *Escherichia coli.* A recombinant protein, named rTcTI, was purified and characterized. The kinetics of the inhibition of the protein were determined and the analysis of recombinant thermostability was performed. The secondary structure was examined using circular dichroism, and the three-dimensional structure was modeled by means of similarity. rTcTI was tested in am in vivo model against larvae of *Helicoverpa armigera* (Hübner) (Lepidoptera: Noctuidae, an important polyphagous pest of agricultural crops worldwide. It accumulates in resistant varieties of cocoa when infected with Mp, being identified as different IT isomorphs, showing a possible form of post-translational regulation to the attack by *Mp*.

## Materials and methods

### In silico analysis of the sequence of TcTI and phylogenetic analysis

The complete nucleotide sequence of the *Theobroma cacao* trypsin inhibitor was obtained from the cocoa EST database (http://esttik.cirad.fr/, accession number KZ0ACR1YI20FM1).

The amino acid sequence was obtained using the Expasy Translation tool. The signal peptide prediction was conducted using the SignalP 4.0 Server^34^. The recognition of conserved domains was done using Pfam (http://pfam.xfam.org/search/sequence)^35^, and analysis carried out with MEROPS (http://merops.sanger.ac.uk/).

The phylogenetic tree was constructed using the Mega 7.0 software, based on the neighbor-joining method. The sequences analyzed for the formation of the phylogenetic tree came from seven gene sequences for the trypsin inhibitor found in the Cirad cacao genomic bank (http://esttik.cirad.fr/). From the alignment analyses, sequences of other homologous proteins with over 80% similarity were considered for the construction of the phylogenetic tree.

To analyze the transcriptional profile of TcTI in *T. cacao* infected by *Mp*, reference files containing the transcripts of *Theobroma cacao* cv ‘Comum’ (Forastero genotype) in FASTA format (GCA_000208745.2_Criollo_cocoa_genome_V2/) were used. These files were obtained from the GenBank database (https://ftp.ncbi.nlm.nih.gov/genomes/genbank/plant/Theobroma_cacao/latest_assembly_versions/) through the RNA Galaxy workbench 2.0 platform (https://rna.usegalaxy.eu), using the Salmon extension^36^. Twenty-one transcripts corresponding to ~ 21 kDa proteins with characteristics of Kunitz-type trypsin inhibitors (KPIs) were selected. The relative quantification of each transcript corresponding to the TcTI gene and its gene family was performed based on public data from ten libraries (five from the control condition and five from the condition of infection by Mp—biotrophic phase) of RNA-Seq data from *T. Cacao*^37^, available at NCBI's SRA (https://www.ncbi.nlm.nih.gov/sra) under number SRA066232. In summary, the identified transcripts corresponded to the infected apical meristem tissues of *T. cacao* 30 days after inoculation with a suspension of *M. perniciosa* basidiospores, representing the green broom stage. In order to find the proteins associated with the analyzed transcripts, BLAST analysis using the BlastX command (https://blast.ncbi.nlm.nih.gov/) was performed and a heat map was generated for visualization of the expression profile of the transcripts using the ComplexHeatmap packages in the R statistical software (R Core Development Team 2013)^38^.

### Molecular cloning of TcTI

The forward primer TcTINdeIF (5′ GGTAGCCAGACATATGGAATCTCCGGTG 3′) and reverse primer (antisense) TcTIXho1R (5′ CCTCCACCCTCGAGCAAGACTCTG 3′) flanked the ORF, excluding the signal peptide, resulting in a 741 bp fragment between bases 115 and 820 of the cDNA. The gene fragment was amplified by PCR using the DNA of the KZ0ACR1YI20FM1 clone from the EST bank from CIRAD (http://esttik.cirad.fr/) as template. This clone was obtained from a library of hybrid seedlings from Papua and New Guinea varieties as a result of the intersection 17/36 × 3–1/3–1^39^. PCR was performed with 30 ng of DNA, 5 µL of 10X reaction buffer, 3 µL of dNTPs (0.2 mmol L^−1^ of each nucleotide), 25 µL of MgCl_2_, 3 mol L^−1^ of each primer, and 0.2 unit of *Taq* DNA polymerase (Fermentas) in a final volume of 50 µL. The amplification cycle consisted of an initial denaturation step of 5 min at 94 °C, followed by 40 cycles of 45 s at 94 °C, 30 s at 54 °C, 1.5 min at 72 °C, and a final extension step of 15 min at 72 °C. The amplified fragment was purified with the PCR Purification Kit (Fermentas) and digested with the enzymes Nde1 and Xho1. The Tcti fragment was inserted into the pET-28a vector (Novagen). The recombinant plasmid was inserted by heat shock into competent cells of the *E. coli* strain (*Rosetta* [*DE*_*3*_]) according to^40^.

### Expression and purification of rTcTI in E. coli

The *E. coli* culture transformed with the recombinant plasmid was grown in LB medium (Luria–Bertani) and incubated at 37 °C under stirring at 200 rpm until OD_600_ 0.5 and 0.7 ηm. The expression was induced by adding 0.4 µmol L^−1^ of IPTG (isopropyl beta-D-thiogalactoside) for 4 h at 37 °C. The cell culture was then centrifuged at 10,000 g, and the precipitate was dissolved in binding buffer and lysozyme (100 μg mL^−1^) for 30 min. Total bacterial extract was ultrasonicated for 20 s interspersed with 30 s of rest on ice using the amplitude parameter of 70% in the ultrasound processor (pGEX 30). This process was carried out until the extract lost its viscosity, producing total membrane disruption. Purification of the protein rTcTI was performed as described by Pirovani et al.^33^.

### Inhibitory activities

#### Activity against porcine trypsin

The trypsin inhibitory assay was performed using BApNA (Nα-benzoyl-D, L-arginine 4-nitroanilide hydrochloride) as a substrate. A total of 10 µl of trypsin solution (0.3 mg mL^−1^ in 2.5 mmol Tris–HCl, pH 7.5) was incubated for 15 min at 37 °C with 60 µL of inhibitor rTcTI solution (0.5 mg mL^−1^) and 120 µL of 50 mmol L^−1^ Tris–HCl buffer, at pH 7.5. The molar ratio of rTcTI versus trypsin was 1:1 for all assays. Reactions began with the addition of 200 µL of 1.25 mol L^−^BApNA solution. The colorimetric results were measured by absorbance at 410 ηm. Residual activity of 100% trypsin was attributed for the control readings (no inhibitor). The activity of the remaining concentrations of the inhibitor was calculated using the controls following equation: Residual activity [%] = [∆ABS410 CI/∆ABS410 SI] × 100, where ∆ABS410 SI corresponds to the change in absorbance at 410 ηm in the absence of the inhibitor, and ∆ABS410 CI corresponds to the change in absorbance at 410 ηm in the presence of the inhibitor.

### Determination of the inhibition constant [Ki]

The inhibition constant [Ki] was calculated using different substrate concentrations. The assay was performed in quadruplicate for 0 (control), 66.66, and 133.33 ηmol L^−1^ of the final concentrations of inhibitor rTcTI against 10 µg of porcine trypsin (Sigma). The reactions were incubated for 15 min at 37 °C and the BApNA substrate was added to obtain the final concentration of 0.04–0.42 ηmol L^−1^ for a final volume of 240 µL. The microtiter plate was read in a SpectraMax Paradigm reader at 410 ηm, monitored by SoftMax Pro 6.3 (Molecular Devices).

The data were fitted to the linear model by double-reciprocal plot. For determination of Ki, the following equation, was used: Ki = [I]/[Km /Kmi] -1, where Kmi corresponds to the average of the apparent Km for the two inhibitor concentrations and Km is the value of the Michaelis–Menten constant without the presence of the inhibitor.

### Test of thermostability of rTcTI

Aliquots of 7 μmol L^−1^ of the recombinant protein rTcTI were incubated at temperatures of 40, 50, 60, 70, 80, and 90 °C for 10 min. Aliquots of 20 μl of protein from each thermal treatment were placed in triplicate in an ELISA plate and 10 μl of porcine trypsin (Sigma©) at concentration of 0.5 mg mL^−1^ was added and incubated at 37 °C for 15 min. Afterwards, 200 μl of BApNA 1.2 mmol L^−1^ was added to all treatments. Chromogenic substrate hydrolysis followed the same parameters previously calculated for the inhibition curve. The residual activity calculations at different temperatures were performed by the same method used for the inhibition curve with the control reaction, using the same intensity of heat treatment without the inhibitor, stipulated as 100% inhibitory activity.

### Structural analysis

#### Circular dichroism (CD)

The rTcTI protein was subjected to CD analysis in a J-815 spectropolarimeter (JASCO). The TcTI protein was purified from the *E. coli* extract and was subsequently dialyzed in phosphate buffer at 50 mmol L^−1^, pH 7.4, to remove the salt solution, thereby avoiding any interference in the analysis. To identify the presence of secondary conformations, a scan spectrum of 190–250 nm was used in 1 mm quartz cuvettes. Data were collected with a scan rate of 50 nm min^−1^ and 0.5 nm range. Readings were performed at 26 and 96 °C after 5 min incubation under the same temperatures, and the average of three consecutive measurements was used for the analysis. The percentage of the secondary structure based on the CD spectrum was calculated with the K2D3 software^41^.

### Molecular modeling of TcTI

The structure of the trypsin inhibitor from *Theobroma cacao* was inferred using the Swiss-Model server (http://swissmodel.expasy.org/)^42^. NCBI BLAST search of the Protein Data Bank (PDB), *Murraya koenigii* MkTI (PDB ID: 3IIR)^43^, showed identity with TcTI, so it was selected as the template to construct the TcTI structure. The quality of the TcTI structure was checked using PROCHECK 3.4 and Anole in the Rampage server (http://mordred.bioc.cam.ac.uk/~rapper/rampage.php) was used to evaluate and assess the accuracy of the model^44^.

### Bioassays with T. cacao and the causative agent of witches' broom disease [WBD]

This work is part of the project entitled "Proteomic analysis of proteases in the development of witches' broom in the Theobroma caco-*Moniliophythora perniciosa* interaction", registered under number A8AD1C0 in Brazil’s National System for the Management of Genetic Heritage and Associated Traditional Knowledge (SisGen), according to Law 13,123/2015. In addition, all biological material used in this study was handled following the institutional regulations of the Executive Commission of the Cacao Crop Plan (Comissão Executiva do Plano da Lavoura Cacaueira—–CEPLAC) and the Embrapa Environmental Research unit (Embrapa Meio Ambiente), and these species are not listed in the Policy Statement on Research Involving Species at Risk of Extinction (IUCN). All experiments were performed in accordance with relevant guidelines and regulations. The accumulation of IT inhibitors in cocoa meristems infected by the fungus *M. perniciosa* was analyzed. Two varieties of cocoa were selected for the experiment, Catongo and TSH1188, susceptible and resistant varieties, respectively. After 30 days of cocoa seed germination, 240 seedlings were selected of each variety and kept in the greenhouse at the CEPLAC premises.

A total of 120 seedlings of each variety were separated for inoculation with a 2,105 × mL^−1^ of basidiocarp suspension (*M. perniciosa*)^45^ and maintained for 24 h in a humid chamber at 25 °C. This acclimatization allowed the germination of *M. perniciosa* spores, their penetration and consequent plant infection.

The meristems (2–3 cm segments) were collected at 1, 5, 45 and 60 days after inoculation. Approximately 10–20 meristems were collected for each stage. The extraction of total proteins followed the protocol described by Pirovani et al.^46^. The protein extract was measured with the 2D Quant Kit (GE Healthcare) according to the manufacturer's manual of recommendations. The protein extract concentrations were normalized for the analysis of IT accumulation by the western blot technique^40^, and fragments were quantified by the Gel.Quant 3.1 software. The protein extracts of the different treatments were normalized for analysis on the 2D SDS-PAGE gel, as described by Santos et al.^47^.

### Bioassays against larvae of Helicoverpa armigera

The insects were obtained from the colony maintained at the Costa Lima Quarantine Laboratory, Embrapa Meio Ambiente, reared with artificial feed according to the method described by Vilela et al.^48^. For biological tests, *Helicoverpa armigera* larvae at 7 ± 1 days of age and 2 h of starvation were used. Each replicate was composed of one larva placed individually in a Petri dish (diameter = 9 cm) and maintained in a growth chamber at 27 ± 1 °C, 70 ± 5% relative humidity and 12:12 photoperiod (L: E). There were 13 replicates per treatment. Soybean leaves were cleaned and immersed in a solution of 0.1 mg mL^−1^ rTcTI in 10 mmol L^−1^ phosphate buffer at pH 7.2 with Triton X-100 (0.01% v/v). Treated leaves were allowed to dry at room temperature and were offered to the *H. armigera* larvae for 24 h. The leaves with rTcTI and control treatments (10 mmol L^−1^ phosphate buffer, pH 7.2 with Triton X-100 [0.01% v/v]) were removed and the larvae were all maintained on artificial feed (the same utilized to maintain the insect colony) under controlled conditions. The larvae were weighed before treatments and one, three, and seven days after the bioassay, and the mortality was measured until the death of all larvae. The percent reduction in weight gain was calculated according to the method described by Halder et al.^49^ using the following equation: Reduction in larval weight [%] = [[Larval weight of control—Larval weight of treatments TcTI] Larval weight of control^−1^]] * 100. The amount of leaf surface consumed was determined using a LI-COR leaf area meter (LI-3100A; LI-COR Biosciences, Lincoln, NE, USA) before and after 24 h of exposure to the larvae. Analysis of variance (ANOVA) and the Scott-Knott test were applied to data on leaf area consumption, larval weight and mortality, using the Sisvar software with *p*-value < 0.05.

### Collection of biological samples

The authors declare that all biological material used in the study was collected and treated in accordance with the institutional and national norms of theExecutive Commission of the Cacao Crop Plan (Comissão Executiva do Plano da Lavoura Cacaueira—CEPLAC) and the Embrapa Environmental Research unit (Embrapa Meio Ambiente).

## Results

### TcTI sequence analysis

**Figure 1:**
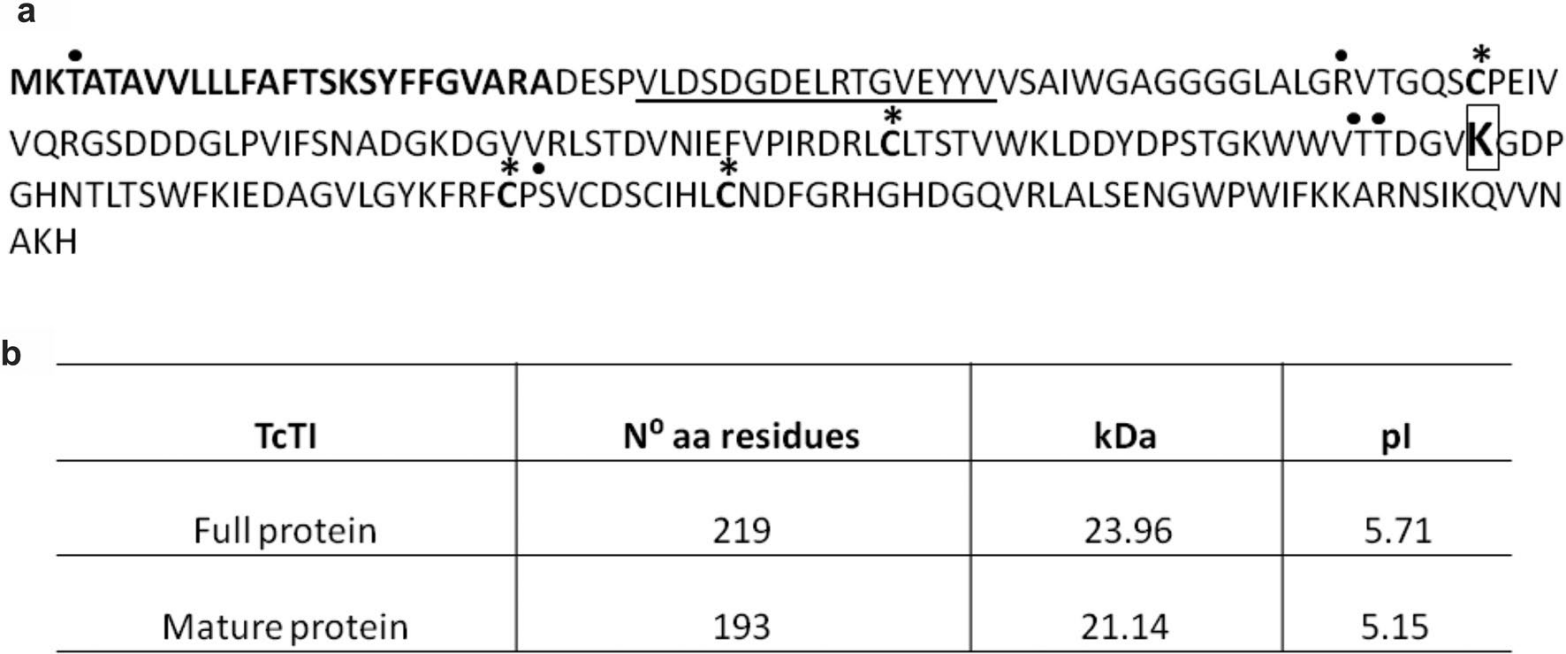
The amino acid sequence of the TcTI protein. (**a)** The signal peptide is in bold. The underlined area represents the Kunitz conserved domain. The inhibitory lysine residue is presented in the box. The black dots indicate possible glycosylation sites. The asterisks in the cysteine residues are associated with disulfide bridge formation. (**b)** Prediction of TcTI parameters. Number of amino acids, theoretical size, and isoelectric point.

Sequence analysis of the trypsin inhibitor identified in the EST cocoa library^39^ revealed an open reading frame sequence of 660 bp encoding a protein with 219 amino acids (Fig. 1b). The analysis also predicted molecular weight and isoelectric point of 23.96 kDa and 5.71, respectively. The molecular weight and isoelectric point of the mature polypeptide without the signal peptide were 21.14 kDa and 5.15, respectively. The analysis of the predicted amino acid sequence of TcTI using the SignalP 4.0 program predicted the presence of a signal peptide with a cleavage site between residues A26 and D27 (Fig. 1a). The protein without the signal peptide showed four possible sites of O-glycosylation, corresponding to T61, T28, T29, and S158 (Fig. 1a). Another analysis using the Pfam program showed that the Kunitz-type protease inhibitor (KPI) domain comprised 20 amino acids and was located between amino acids V21 and V50.

**Figure 2:**
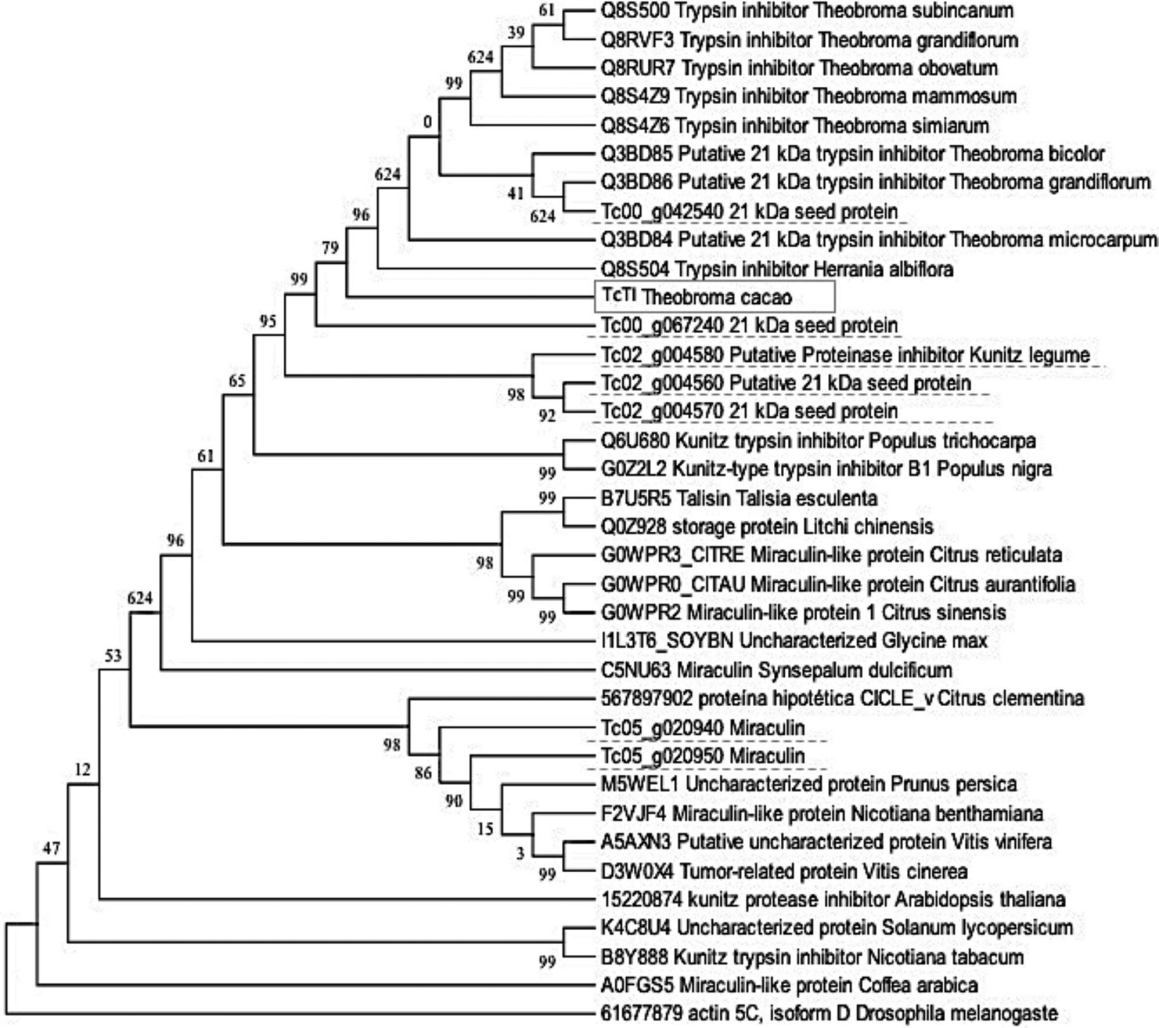
Phylogenetic tree built according to the similarity of TcTI with other inhibitors. The square contains the TcTI. The other genes for the Kunitz-type trypsin inhibitor found in the cacao genomic bank are underlined.

Phylogenetic cluster analysis with the protein sequence of other plant groups showed that the genes for cocoa trypsin inhibitors were grouped into three different branches (Fig. 2). The TcTI protein showed high similarity with inhibitors of species of the same genus *Theobroma* and with a similar genus, *Herrania.* The most similar protein in the cacao genomic bank with TcTI was related to the Tc00_p067240 locus and was slightly more distant from the Tc00_g042540 locus, but in the same branch (Fig. 2). The Tc02 chromosome gene products were grouped in the same branch, showing 99% identity and were closer to TcTI than the Tc05 chromosome gene products. The products of the genes referring to miraculin (Tc05_g020940 and Tc05_020950) located on the Tc05 chromosome in the cocoa genome were grouped in a branch distant from the other cocoa genes. Furthermore, TIs of the Kunitz miraculin type of other species were grouped with the genes related to the cocoa miraculin, showing high similarity due to the conserved characteristics of their sequences.

### Expression and purification of recombinant protein (rTcTI)

The full-length cDNA of 741 bp was obtained and cloned in the *Rosetta DE*_*3*_* E. coli*. The translated polypeptide of 219 amino acids excluded a signal peptide of 26 amino acids. Most of the recombinant protein was detected in the soluble fraction from the bacterial extract. The apparent molecular weight was found to be about 23 kDa. The protein was purified with 100% homogeneity from the soluble fraction of the bacterial extract by means of histidine tail affinity chromatography (HisTag) from the sequence present in the expression vector.

### Inhibitory properties and Ki determination of rTcTI

**Figure 3:**
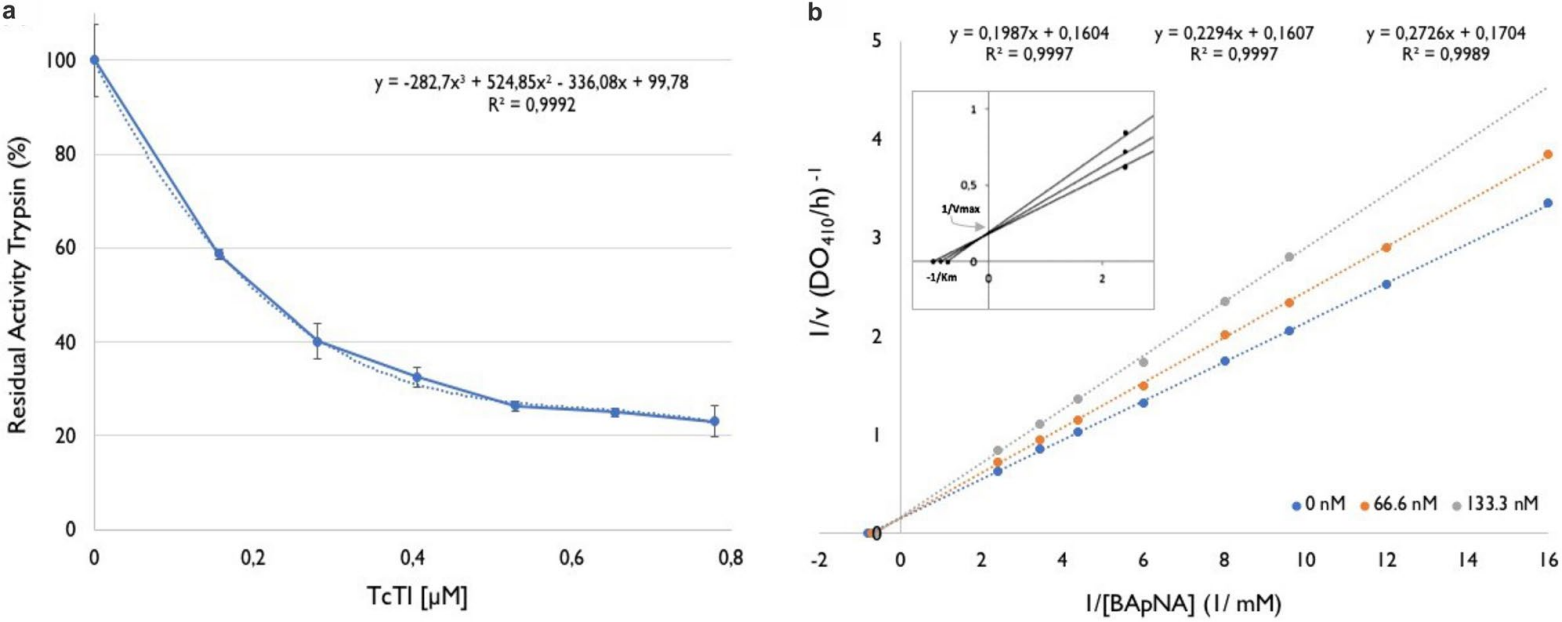
rTcTI inhibits porcine trypsin. (**a)** Residual trypsin activity decreases linearly with increasing concentration of rTcTI. (**b)** Determination of Ki based on Lineweaver–Burk plot. Double-reciprocal model for the inhibition of rTcTI on trypsin, in competition with the substrate BApNA. TcTI at three different concentrations: 0, 66.6, and 133.3 ηmol L^−1^.

The rTcTI produced in a heterologous system in this study was active because residual trypsin activity was markedly reduced with increasing concentration of rTcTI in the reaction medium (Fig. 3a). The trypsin activity decreased to 70% at the concentration of 0.5 mol L rTcTI. An increase in concentration from 0.5 to 1.2 μmol L^−1^ of rTcTI reduced the residual activity to approximately 12% (Fig. 3a). The determination of the maximum reaction velocity (Vmax) and Michaellis-Menten constant (Km) was necessary to calculate the inhibition constant, Ki. Vmax values were 6.23 mmol L^−1^ min^−1^ in the absence of the inhibitor and 6.22 and 5.86 mmol L^−1^ min^−1^ in the presence of the inhibitor (rTcTI) at concentrations of 66.6 and 133.3 ηmol L^−1^ rTcTI, respectively. According to the double-reciprocal model, the curves showed a very similar intersection point on the 1/Vo axis, indicating that the inhibitor did not affect the Vmax values. The inhibition constant, Ki, determined according to the double-reciprocal model was 4.08 × 10^–7^ mol L^−1^ for porcine trypsin in the presence of the substrate BApNA (Fig. 3b).

### Thermostability studies of rTcTI

**Figure 4:**
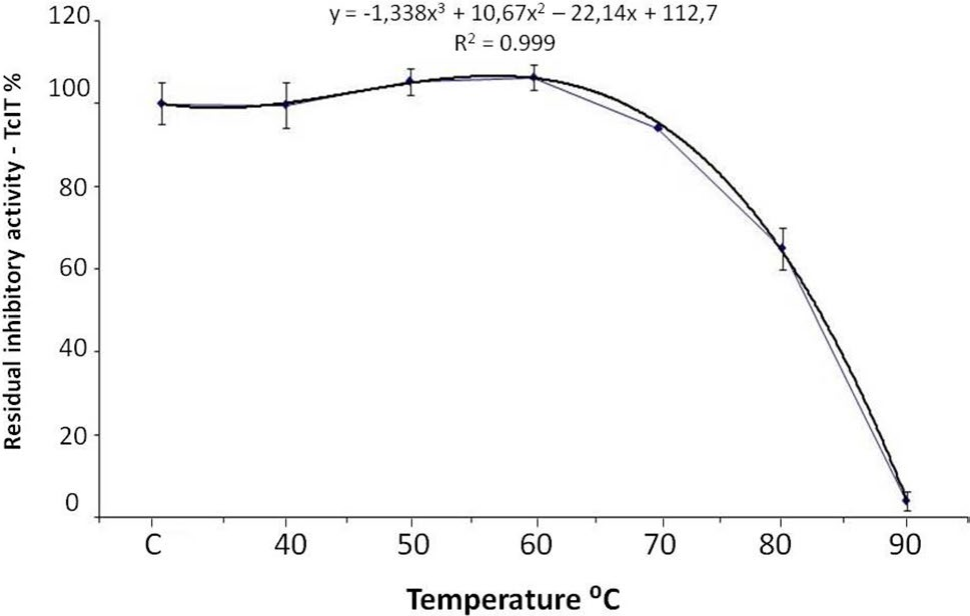
Thermostability of rTcTI at different temperatures. On the X axis, C represents the control reaction performed at 37 °C. The Y axis represents the percentage of rTcTI inhibitory activity in comparison with the control reaction.

The rTcTI was subjected to thermal stability testing by incubation for 10 min. at temperatures of 40–90 °C. The rTcTI showed tolerance to heat treatment up to 60 °C. The treatment at 70 °C induced a decrease in the percentage of inhibition, but it still retained over 90% of its inhibitory activity. Incubation at 80 °C promoted a drop of 40% in its inhibitory activity (Fig. 4). The rTcTI completely lost its inhibitory capacity when treated at 90 °C, indicating complete protein denaturation.

### Analysis of the secondary structure of TcTI

**Figure 5:**
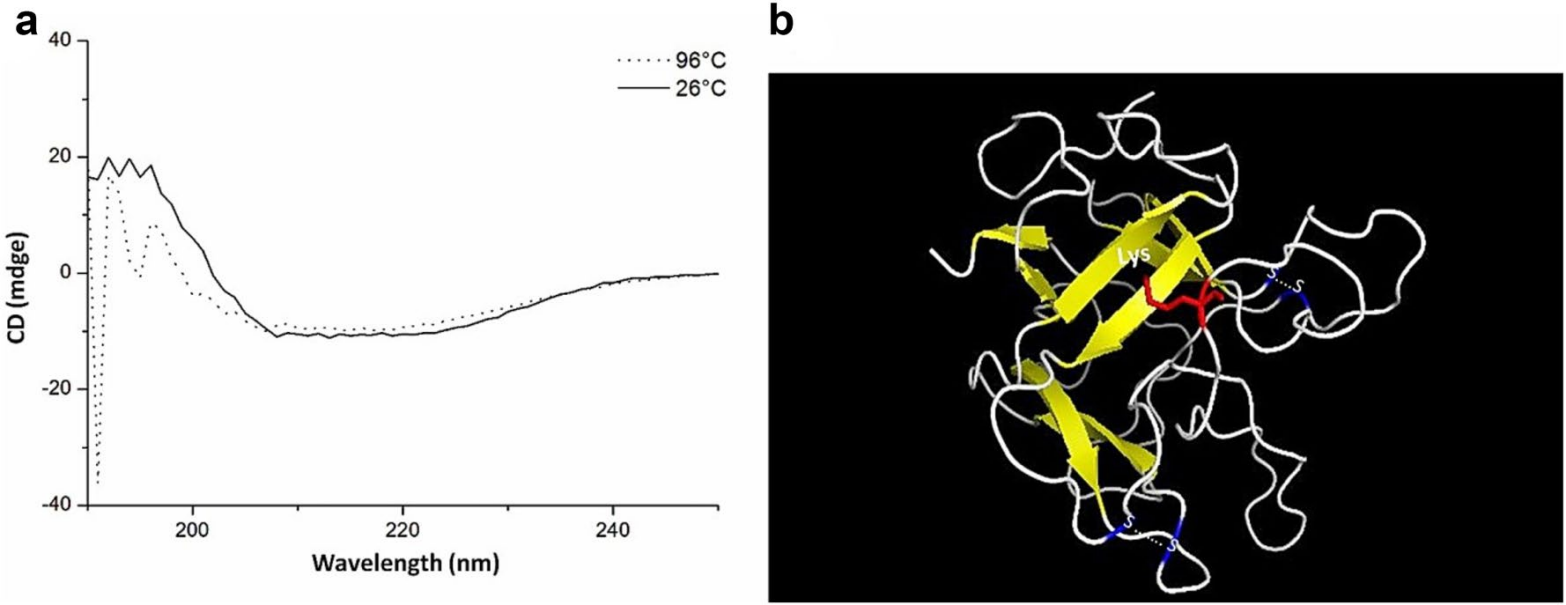
TcTI is rich in loops. (**a)** Circular dichroism analysis of rTcTI protein. The sweep was between 150 and 250 ηmol L^−1^ wavelengths, and the dotted line and the continuous line show the scan profile at 96 °C and 26 °C, respectively. (**b)** Three-dimensional model of *T. cacao* TcTI by homology to mold 3IIR. The protein has antiparallel leaves-β [yellow] and predominance of loops [gray]. The two bisulfide bridges are marked in blue, indicating the cysteine residues. Inhibitory residue [lysine] is marked in red.

The structural analysis of rTcTI by CD measurements showed a predominance of irregular secondary structure, with absence of the α-helix and the presence of β-strands. The presence of β-strands was indicated by the negative peak at approximately 209–220 ηm (Fig. 5a). This analysis was performed at two different temperatures, 26 °C and 96 °C, to detect possible variations in the rTcTI structure during denaturation by heat. The rTcTI spectrum was similar for both treatments, having the same type of negative peak at 220 ηm, indicated by the dotted line graph for the treatment at 96 °C (Fig. 5a). The spectrum value obtained by CD was measured by the K2D3 server, indicating that the secondary structure of the TcTI has 37.8% beta sheet, 35.2% random coil and 8.8%, alpha helix.

The 3D model was predicted by comparative analysis with other inhibitors deposited in the Protein Data Bank. For the creation of the three-dimensional model, the 3IIR mold structure was used, with an identity of 44.8% (*Murraya koenigii*)^43^. According to the Ramachandran plot (Supplementary Fig. 1), 96.9% of residues are in energetically favorable regions. TcTI showed 12 regions of antiparallel β-strands and a small torsion in α-helices. This protein presented an inhibitory loop (Lys), as indicated in Fig. 5b.

### Identification of PIs in 2D gel of T. cacao genotypes against WBD infection

**Figure 6:**
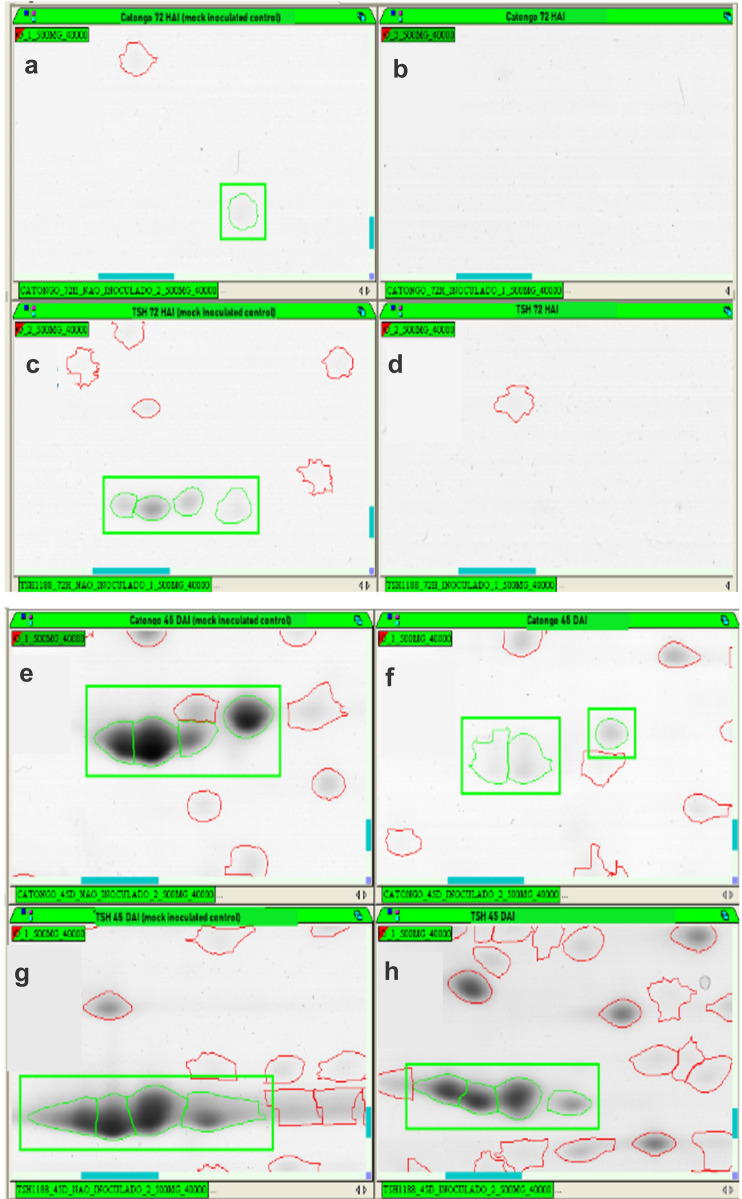
Representative 2D gels of PI protein spots obtained from shoot tips of Catongo and TSH1188 inoculated and non-inoculated [mock inoculated control] at 72HAI and 45DAI post-infection with *M. perniciosa*. Total protein extract [500 μg] were focused on IPG strips [13 cm], with pH ranging from 3 to 10. NL separated by SDS-PAGE [12.5%] and stained with CBB G-250. The green highlight indicates putative PI isoform spots. (**a)** Catongo 72 HAI non-inoculated [mock inoculated control]. (**b)** Catongo 72 HAI. (**c)** TSH1188 72 HAI non-inoculated [mock inoculated control]. (**d)** TSH1188 72 HAI. (**e)** Catongo 45 DAI non-inoculated [mock inoculated control]. (**f)** Catongo 45 DAI. (**g)** TSH1188 45 DAI non-inoculated [mock inoculated control]. (**h)** TSH1188 45 DAI.

PIs temporal dynamics involved in the WBD (witches’ broom disease) development using two-dimensional gel electrophoresis analysis comparing inoculated and non-inoculated (mock inoculated) shoot tips of TSH1188 and Catongo genotypes, in different stages of infection –72 h after inoculation (72 HAI) and 45 days after inoculation (45 DAI)—were characterized. Differential metabolism of PI accumulation was investigated between genotypes. Fourteen spots corresponding to PIs in TSH1188 (1 spot at 72 HAI and 13 at 45 DAI) and 10 in Catongo (1 at 72 HAI and 9 at 45 DAI) (Supplementary Fig. 3 and Supplementary Table 1) were identified. Overall, TSH188 had more spots identified and accumulated compared to Catongo. Both genotypes showed less spot identification and down accumulation of PIs at 72 HAI (Fig. 6a,b,c,d and Supplementary Fig. 3). However, at 45DAI the identification of TIs was higher in both genotypes. Five TI isoforms were down accumulated in TSH1188, but in lesser intensity than in Catongo, which in turn showed high down accumulation of TI isoforms (Fig. 6e,f,g,h and Supplementary Table 1). It is interesting to note that TSH1188 also showed upward accumulation of 7 TIs at 45 DAI, while Catongo showed only down accumulation of these proteins. Among those upward accumulated TIs from TSH1188, five putative miraculin-like proteins were encountered. BLAST analysis of proteins also showed similarity with TIs of *T. cacao*. All identified PI spot sequences and further proteomic information can be found in Supplementary Table 1.

### Bioassays with T. cacao and the WBD pathogen

**Figure 7:**
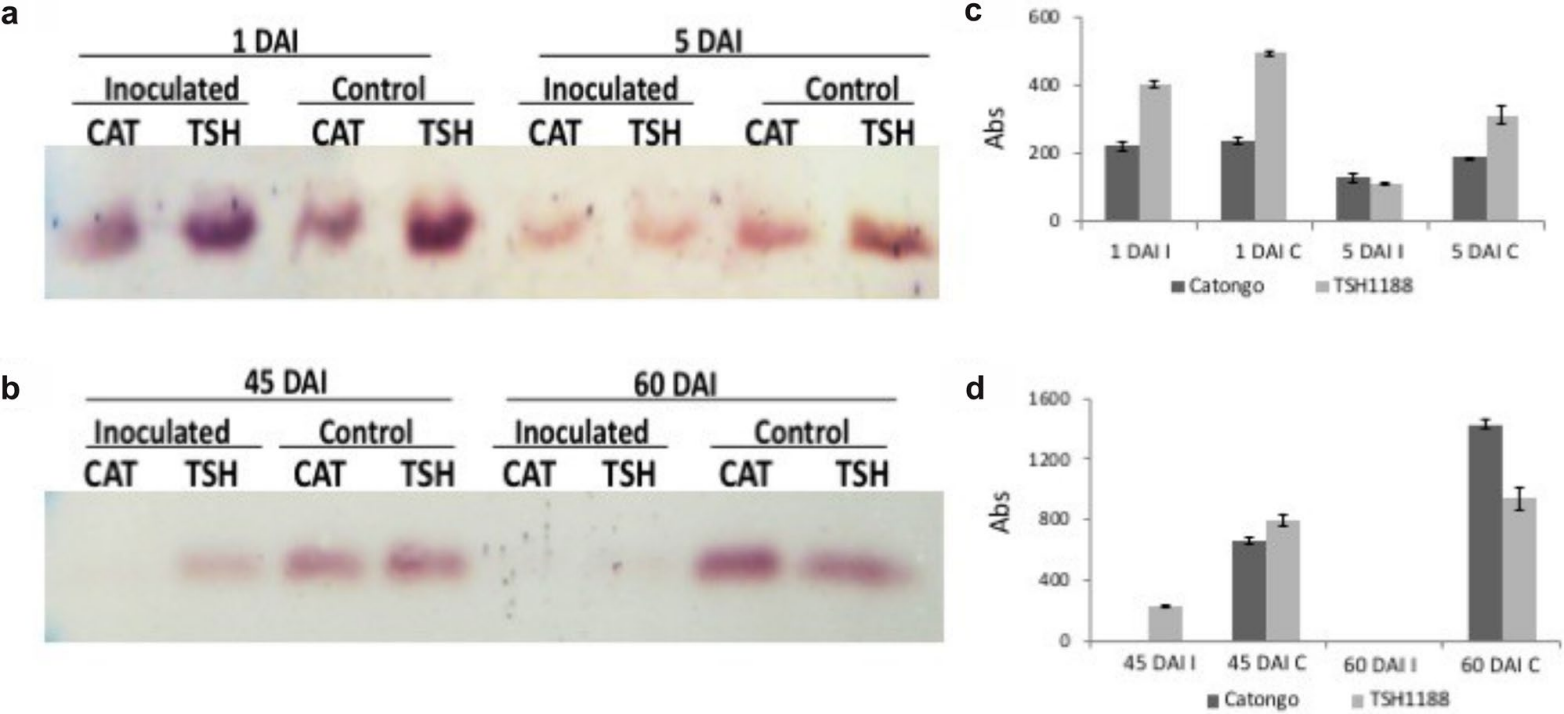
Immunodetection of trypsin inhibitors in cocoa meristems of the TSH1188 and Catongo varieties. (**a)** Initial stages 1 and 5 DAI [days after infection]; (**b)** Final Stages 45 and 60 DAI indicate the periods of infection with *M. perniciosa* and its controls, CAT; Catongo [susceptible variety], TSH; TSH1188 [resistant variety], (**c,d)** Graphs of intensity of the protein bands referring to the different treatments, quantified with the Gel.Quant. [I—inoculated and C- control].

The analysis of the protein profile of meristems showed the difference in proteins expressed under different treatments. The fragment of approximately 23 kDa presented a more intense variation of expression in comparison with the others, being present in both the initial and final stages after inoculation (Fig. 7a,b and Supplementary Fig. 4). This band corresponded to the trypsin inhibitor. In the final stages, the difference in the protein profile in the infected and control treatments was more accentuated compared to the initial stages (Fig. 7b).

The accumulation of the trypsin inhibitor was analyzed in meristems at different times after inoculation with *M. perniciosa*. In the initial stages of infection, at 1 DAI (days after inoculation), there was greater accumulation of inhibitors in the infected treatment for the resistant variety (TSH1188), in contrast to the susceptible variety (Catongo), in which there was a decrease in the accumulation of this inhibitor (Fig. 7c).

In the interval of five DAI, the inhibitor accumulation was lower for both varieties, but for the control treatment in TSH1188, the inhibitor was more abundant according to the western blot test. In Catongo, the accumulation of the inhibitor in the initial stages of infection and its respective controls were lower, maintaining the basal expression. For the final stages of infection, between 45 and 60 DAI, the abundance of the inhibitor decreased considerably in comparison with the control treatments (Fig. 7b,d).

At 60 DAI, considered an advanced stage of the disease, it was not possible to detect significant accumulation of these inhibitors by the western blot technique. This occurred for both varieties. It was not possible to detect the presence of the inhibitor at 45 DAI in the Catongo variety under the experimental conditions, but slight accumulation of the inhibitor was detected in TSH1188.

### Comparison of the transcriptional profile of the KPI gene family

**Figure 8:**
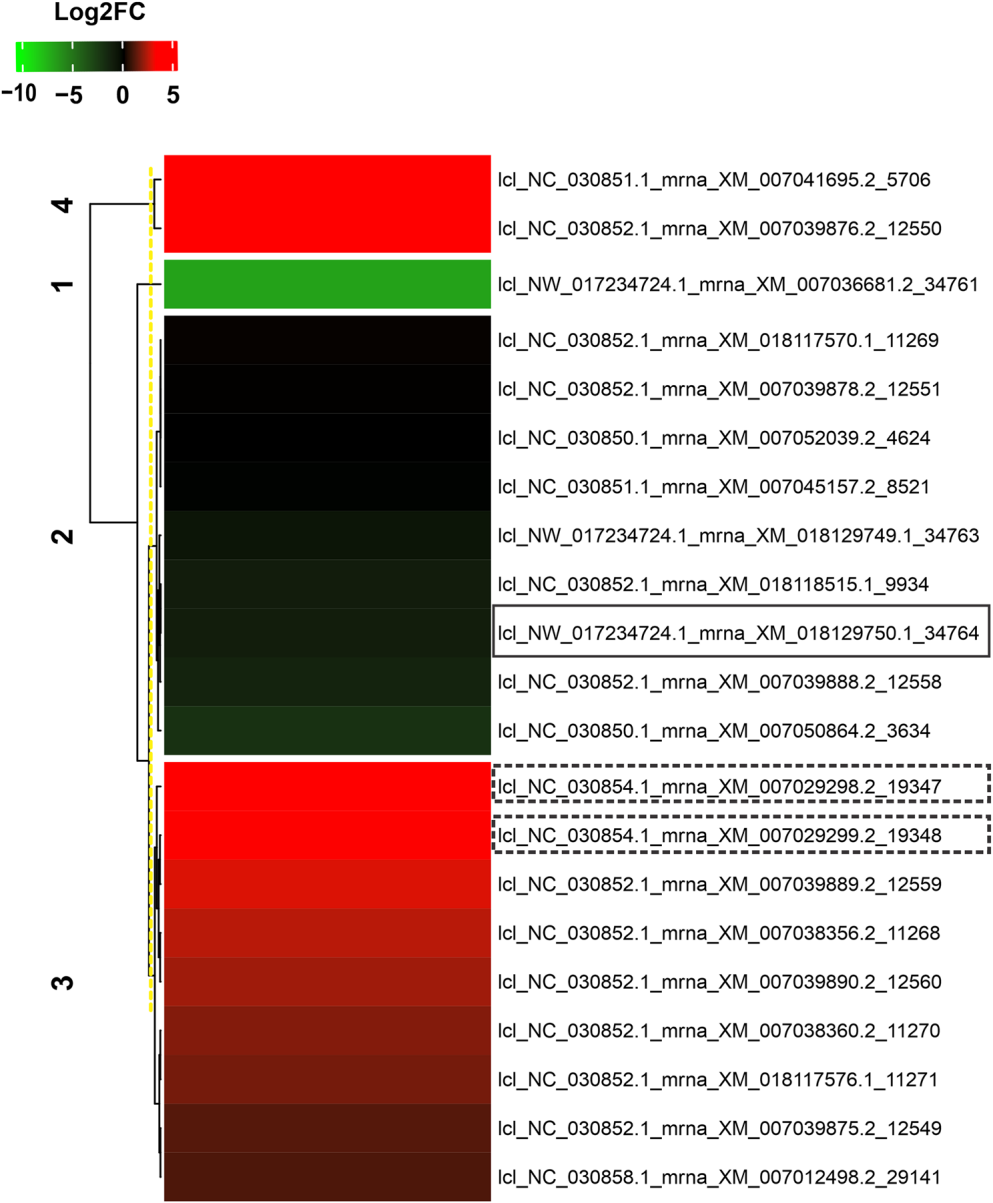
Heat map based on the Pearson correlation coefficient represents the differential accumulation of transcripts that correspond to ~ 21 KDa proteins, including TcTI [highlighted by continuous line] and Miraculin [highlighted by dashed line]. In green, down-regulated transcripts; in red, up-regulated transcripts, related to the Log2FC scale of – 10 and 5, respectively.

To determine the expression pattern of the gene family of the trypsin inhibitor, the relative quantification of 21 transcripts encoding ~ 21 kDa proteins with identity and coverage above 96% and 50%, respectively, was performed (Supplementary Table 2). Based on the results of BLAST X, compared to the findings of Gesteira et al.^29^, the transcript lcl_NW_017234724.1_mrna_XM_018129750.1_34764, corresponded directly to the TcTI protein (EOY21251.1) with 98.63% identity, 66% coverage and 1E-145 e-value. Most transcripts from the gene family of the trypsin inhibitor were positively regulated in the biotic stress condition (Fig. 8). However, the transcript corresponding to TcTI (lcl_NW_017234724.1_mrna_XM_018129750.1_34764) had a repressed expression profile. Some of these transcripts with up-regulated expression corresponded to miraculin (lcl_NC_030854.1_mrna_XM_007029298.2_19347 and lcl_NC_030854.1_mrna_XM_007029299.2_19348) protein accumulated in the resistant genotype, TSH1188 of the present study.

### Assessment of the effect of TcTI on development of H. armigera larvae

**Figure 9:**
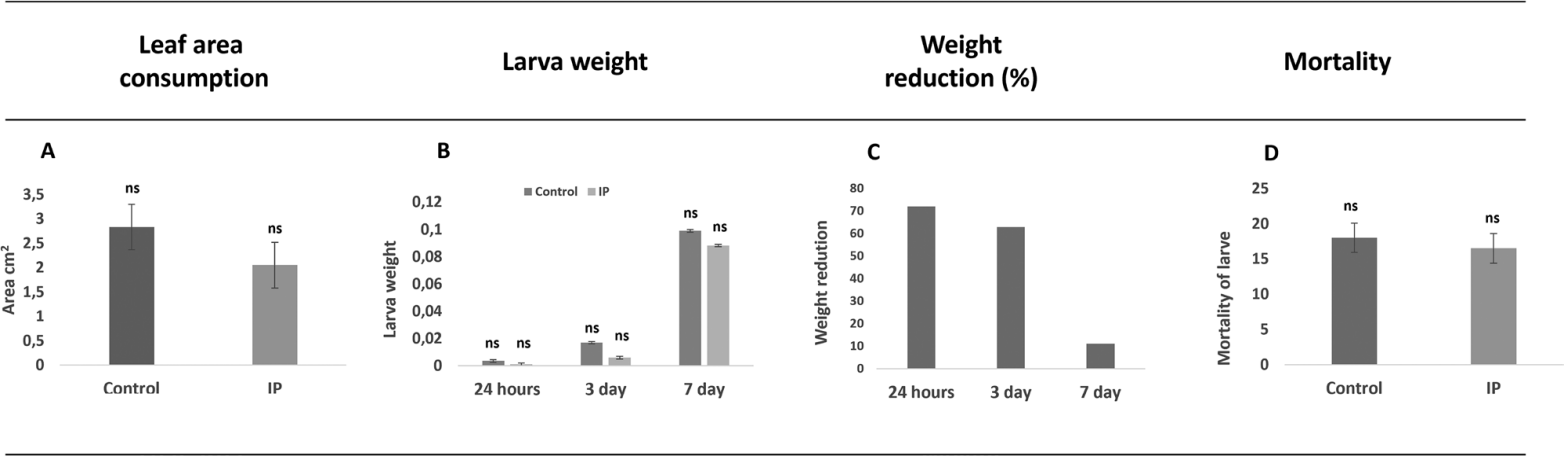
Effect of purified rTcTI against larvae of *Helicoverpa armigera*. (**A**) Average of leaf area [cm^2^] consumed by the larvae. (**B**) Larval weight in the treatment rTcTI and control treatment. (**C**) Percentage reduction of the increase in body mass for the larvae that ingested the rTcTI compared to the control larvae. (**D**) Mortality of *H. armigera* in the second larval instar, approximately 25 days from hatching to death.

*Helicoverpa armigera* larvae were fed with soybean leaves sprayed with rTcTI at 1 mg mL^−1^. Among the larvae that ingested the protease inhibitor (rTcTI), 33.3% died in the second larval instar while only 8.3% of individuals in the control group died in this same larval instar (Fig. 9d). The reduction of weight of the larvae treated with the inhibitor occurred especially during the first 24 h. The number of larvae fed with the rTcTI-treated leaves decreased compared to the larvae in the control treatment (Fig. 9b). The larvae of the rTcTI treatment had 71.9% reduction in weight gain compared to the larvae in the control group. Three days and seven days later, the larvae that ingested IP decreased by 62.9% and 11.0% in terms of body mass gain, respectively, compared to those of the control treatment (Fig. 9c). The no-choice bioassay showed that the rTcTI did not interfere with the larval consumption, since there was no significant difference in the average leaf area consumed by the larvae of the control treatment compared to those treated with rTcTI according to the Mann–Whitney test at 5% probability (Fig. 9a).

## Discussion

### In silico analysis indicated that TcTI is a Kunitz-type inhibitor

The study of cocoa trypsin inhibitors is of particular interest for investigation of plant-pathogen interactions in which the trypsin inhibitor gene has been identified as being differentially expressed during the *Theobroma cacao*–*Moniliophthora perniciosa* interaction, which causes the witches’ broom^29^. In Brazil, *M. perniciosa* is responsible for drastic production and yield losses of cocoa beans in the south of the state of Bahia^50^. The trypsin inhibitor sequence fragment detected in EST libraries of the interaction *T. cocoa–M. perniciosa*^29^, was used to identify the complete inhibitor sequence in the cacao EST database (http://ESTtik.cirad.fr/) ^39^ The analysis of conserved domains identified a region related to the family of trypsin inhibitors of the Kunitz type (Fig. 1a).

The complete TcTI sequence of cocoa has an estimated molecular weight of 219 amino acids at 23.9 kDa, similar to most Kunitz-type inhibitors, which have a mass of 18–24 kDa^28^. Although TcTI has six cysteine residues, four of them occupy conserved regions for the Kunitz family, forming two sulfide bridges. This shows that the TcTI structure is similar to other Kunitz-type inhibitors. In addition, the inhibitors that have more than two disulfide bridges and more than four residues of cysteine are grouped in the same category due to the similar protein structure^51^. This suggests that the additional sulfide bridges do not promote any drastic modification of their own tridimensional structure, which has been demonstrated by means of the tridimensional model, also seen in the PDB 3D model (3iir). The latter presents two regular disulfide bridges of the Kunitz members and contains seven cysteine residues in its formulation^43^.

When analyzing the sequence referring to the Kunitz domain of TcTI, high similarity was found of inhibitors of species of the same genus (Theobroma) with species of the genus *Populus*. The library described by Argout et al.^52^ shows that *Theobroma cacao* shares some families of genes with *Populus trichocarpa*. Another similarity was noticed for the protein miraculin, which acts by limiting cell damage in conditions of biotic stress. This protein has been identified in witches' broom-resistant cocoa genotypes^47^. Similarity was also noted with sporamin, classified as a TI Kunitz protein, present in sweet potatoes. It is a vacuolar storage protein whose gene levels are highly regulated in response to biotic and abiotic stresses^53,54^. This high similarity was confirmed using the ExpasyBlast tool with the sequence of TcTI and other proteins related to the cocoa trypsin inhibitor with TIs of other species. These analyses showed that although these genes are very similar, small variations exist, so they need to be grouped in different clusters (Fig. 2).

### rTcTI accumulates in soluble E. coli extract and exhibits competitive-like inhibition

Eukaryotic proteins generally accumulate in insoluble extracts of *E. coli* for different reasons, such as incorrect folding, failure of the post-translational modification or problems in DNA coding. Despite the fact that the Tcti ORF showed 28.6% rare codes for *E. coli* (Supplementary Fig. 2), the expression of rTcTI was analyzed using *Rosetta* [*DE*_*3*_], a specialized strain with optimized tRNA for rare codons. This strategy was successful since rTcTI accumulated abundantly in the soluble *E. coli* extract and the purified protein was active against porcine trypsin, as shown by the inhibitory assay (Fig. 3a). Other protease inhibitors from cacao have also been successfully expressed in the *E. coli Rosetta* [*DE*_*3*_] strain^33,55,56^.

With a concentration of 0.25 mol L^−1^, rTcTI presented 60% inhibition of porcine trypsin activity. One TI from a scorpion presented a single curve highly similar to the plant-like inhibitor obtained for rTcTI, but the concentration of the inhibitor was tenfold greater than the one analyzed for rTcTI^57^.

The inhibition shown by rTcTI is of the competitive type, since the intersection of the curves occurred on the ordinate axis, indicating changes in the Km values and minimal changes in the Vmax values (Fig. 3b). This is a usual characteristic of these inhibitors from the Kunitz family, indicating that a direct interaction with the catalytic site of the enzyme might exist, the same site that binds to the substrate. rTcTI seems to be a powerful trypsin inhibitor with regard to its Ki value (4.08 × 10^–7^ mol L^−1^), indicating high affinity between rTcTI and swine trypsin. Some Kunitz inhibitors are considered good candidates for strong interaction with enzymes that present lower Ki values and a larger inhibitory capacity, such as the case of Kunitz ITs—Soy Glicine Ki (3.2 × 10^–9^ mol L^−1^)^58^, *Trigonella foenum-graecum* Ki (3.01 × 10^–9^ mol L^−1^^59^, *Entada acaciifolia* Ki (1.75 × 10^–9^ mol L^−1^)^60^, and *Pithecellobium dumosum* Ki (5.7 × 10^–10^ mol L^−1^)^5^. These inhibitors can present even lower Ki values for proteases from insect pests’ digestive enzymes, and these interactions may be even more specific^61^.

### rTcTI is stable at temperatures up to 60 °C

The rTcTI protein presented moderate stability at high temperatures, as shown by the thermal effect study (Fig. 4). The elevated inhibitory activity was present until 70 °C, and in the 10 min of treatment it increased to 80 °C, which revealed a minimum of 10% of its inhibitory capacity. A similar profile was found for the trypsin inhibitor of *Vigna radiata*, which maintained stable activity until 90 °C. The activity decreased stepwise with increasing time since hatching^62^. The *Cassia grandis* CgTI inhibitor also showed high thermostability at 60 °C, maintaining 100% of its inhibitory activity, followed by only slight activity reduction at 80° C^63^.

The thermostability of rTcTI may be related to structures that are partly random coil (Fig. 4). The disordered region to promote greater flexibility of the protein, with a relatively low loss of activity until to occur by chance may promote greater flexibility of the protein, with a relatively low loss of activity until 60 °C. The presence of disulfide bonds in the primary structures of the protein may contribute to this tridimensional structural stability^64^.

### TcTI has a secondary structure rich in non-ordered regions

The TcTI protein has 5.4% α helix and 33.3% β strand based on the CD spectrum. This indicates that the largest part of the protein does not have a well-defined secondary structure (Fig. 5a). The presence of β-sheets is indicated by the negative pitch in the range of 220 ηm in the CD spectrum (Fig. 5a). Similar spectra are also found for other inhibitors of the Kunitz type^57,65^. As already mentioned, the secondary structure of rTcTI is predominantly composed of random coil arranged by chance and some β-sheet regions. The sweep analysis at 96 °C presented a similar spectrum to the previous sweep at 26 °C, showing that the native protein presented a similar profile to the denatured protein (Fig. 5a). However, the central structured part of the protein is substantially responsible for its activity and is not easily renewed, as shown by its activity loss during the thermal treatment (Fig. 4). These characteristics are noteworthy for a Kunitz-type inhibitor, since they do not have well-established α-helices. Similar structures have been described for other Kunitz inhibitors^59,60,66–68^.

The modeling through homology demonstrated that TcTI has a rich structure of random coil, at 35.23%, and a few structures of β-sheets, with 37.82%, corroborating the CD analyses (Fig. 5). The inhibitory site of the trypsin inhibitor usually contains an arginine (Arg)^69–71^ in TcTI. The active site is located in an inhibitory loop region with Lys 141, similar to inhibitors of the Kunitz types CpTI^72^ and ILTI^73^.

### Transcriptional profile of the KPI gene family operates in the initial stage of Mp infection

The *T. cacao* genome contains a large gene family of trypsin inhibitors of the Kunitz type, whose transcripts were detected in the cDNA library of the interaction between *T. cacao* and *M. pernciosa* by^29^, which correspond to ~ 21 kDA proteins. The differential expression of the 21 selected genes (Fig. 8) can be changed according to the pathogen, inoculation time, stage of infection and plant species^74^. However, having a diversity of KPIs (Kunitz-type protease inhibitor) can also be advantageous for the plant in interacting with pathogens.

Considering that the *Mp* fungus was in the advanced infection phase [biotrophic phase] and the transcript corresponding to TcTI showed negative regulation, this pattern may have been caused by the early expression of trypsin inhibitors, possibly indicating that cell damage occurred at the onset of infection^75^. Additionally, with a transcriptional profile of the TcTI not accumulating 60 days after infection/inoculation (Fig. 8) based on studies by Teixeira et al.^37^ in experiments with cacao genotypes (Catongo and TSH 1108), Santos et al.^47^ characterized the dynamics of proteins involved in the development of WDB disease and found up-regulated proteins related to trypsin inhibitors in the resistant cacao genotype 60 DAI. Gesteira et al.^29^ analyzing ESTs from the same two cacao genotypes 90 DAI and identified more than 30 expressed genes corresponding to trypsin inhibitors in the *Mp*-resistant genotype (TSH 1108).

This discrepancy between the protein profile and transcriptional profile can be explained by post-translational changes undergone by proteins that influence their accumulation^76^. In addition, this difference in transcript-protein expression pattern may also be associated with the variety of cacao used by Teixeira et al.^37^, which differs from the genotypes we used and the studies carried out by Gesteira et al.^29^ and Santos et al.^47^.

### TI isoforms accumulate in the early stages of WB in a resistant cocoa genotype

Gesteira et al.^29^ identified 32 trypsin inhibitor ESTs only for meristems of a resistant variety of *T. cacau* from an accumulative pool of different stages of infection by *M. perniciosa*. According to the immunodetection analysis of *T. cacao* meristems infected by WBD, a variation in the dynamics of differential inhibitor accumulation between contrasting cocoa genotypes was identified (Fig. 7). In the early stages of infection, there is greater accumulation of these inhibitors in the resistant variety TSH1188, such as the initial response to infection by the fungus between 1 and 5 days after exposure. In the most advanced stages of the disease, between 45 and 60 days, the accumulation of these inhibitors decreases significantly. Similar behavior was noticed in the study by Santos et al.^47^, who traced the protein profile of two varieties of *T. cacau*, TSH 1188 and Catongo, the same ones used in the present study, infected by WBD. They found that IPs were differentially expressed in both varieties, but with advancing disease, the resistant variety TSH 1188 presented down-regulated IPs. The same result was observed when the profile of the IPs was analyzed in 2D gels. In addition, it is possible to see different isoforms and IP intensity in the different *Mp* treatments of *T. cacao* (Fig. 7 and Supplementary Table 1).

In resistant cocoa plants, a large amount of hydrogen peroxide (H_2_O_2_) is produced at the beginning of *Mp* infection, which contributes to the control of infection and plant resistance. This response is accompanied by increased expression of some genes, such as Glp (germin-like oxalate oxidase protein from cacao), which acts for the formation of H_2_O_2_, as a temporary defense response of the plant^77^.

Alves et al.^78^ also found differential expression of genes of the GPX family (glutathione peroxidase) in cocoa plants inoculated with *Mp*, at the initial stage of infection. Greater accumulation of PRs was also found from the onset of the disease to 45 days after infection, in the biotrophic phase of the disease^37,47,79^. The phylloplanin gene (TcPHYLL) and other plant defense genes also have their expression up-regulated, which increases the levels of transcripts in cocoa seedling tissues inoculated with the *Mp* fungus, indicating that this gene is related to biotic stress response induced early infection^80^.

Increased expression after inoculation in resistant plants, followed by decreased expression, is a pattern observed regarding defense proteins in *T. cacao* plants, such as in legumain, TcLEG9^81^. Additionally, Alves et al.^78^ also detected increased expression of GPX family genes in the green broom phase in susceptible cocoa plants.

Pirovani et al.^33^ proposed a direct role of cacao cystatins in defense against *Mp*, and also described their action in the development of programmed cell death symptoms. With regard to cystatin, Cardoso et al.^55^ characterized a cysteine (TcCYSPR04), suggesting that within 72 h after MpNEP-plant interaction, there is participation of several isoforms of cysteine-proteases in physiological events in the molecular battlefield of the interaction of *T. cocoa* and *Mp*. However, after the initial phase of infection, with the onset of the biotrophic phase of the disease, a pattern between the pathogen and host is established that can last from 45 to 90 days, characterized as the transition to the saprophytic phase, causing new peaks of expression of defense-related genes. It is thus possible to infer from our data that the pattern of accumulation of PI's followed the gene expression that is commonly observed in defense genes in resistant cocoa genotypes. While for susceptible genotypes there is no increase in H_2_O_2_ production at the beginning of the infection, this occurs later, in the transition to the biotrophic phase of the disease^82^.

Moreover, studies suggest that Kunitz-type trypsin inhibitor genes are in a constant evolutionary process with possible modifications between different genotypes of the same plant species^74,83^. This constant evolution reflects the importance of these proteins in the *T. cacao* x *M. perniciosa* pathosystems, since detection of small structural differences can provide useful information about the specificity and its mechanism of action, resulting in the need to characterize these isoforms to strengthen the biotechnological application of PIs. Another group of inhibitors from the phytocystatin family also accumulated abundantly in the tissue of mature leaves, but only in infected leaves. No significant accumulation was seen by Pirovani et al.^33^. This decrease in inhibitors in cocoa plants may occur due to the response mechanism of cocoa, which activates the programmed cell death signaling pathway, affecting the expression of some proteases, as described for cysteine protease, which increases in infected tissues^46,55^. In meristems under normal conditions, the accumulation of the inhibitor gradually increases during the maturation stages, showing that these inhibitors are part of the natural protection of meristem tissues (Fig. 7b).

However, when we analyzed the PIs accumulated in TSH1188 throughout the development of WBD, five putative miraculin-like proteins were found. It is possible to observe the expression pattern of the transcripts corresponding to miraculin in Fig. 8. They were regulated in *T. cacao* at 60 DAI. Miraculines are glycoproteins related to cell stress, including biotic stress, to limit cell damage, particularly due to their amino acid sequence similarity with Kunitz-type inhibitors^84,85^. Miraculin genes described as Kunitz-type protease inhibitors were identified in studies by Teixeira et al.^37^, a finding that corroborates the idea that the defense responses of *T. cacao* are already induced in the biotrophic phase of the fungus *Mp*, which corresponds to the green broom stage of the disease.

### rTcTI affects larval growth of the H. armigera

rTcTI can inhibit growth of pests, such as *H. armigera*, as shown by feeding studies and tests carried out with rTcTI to evaluate its effects against the larvae of *H. armigera*. These larvae presented lower weight gain when fed with soy leaves with rTcTI (Supplementary Table 3).

In the most advanced stages, after 7 days of life, the larval weight differences were not statistically significant. However, the larval weight gain was less affected by the rTcTI inhibitor. This fact may be related to our feeding of the larvae with rTcTI only in the first 24 h, allowing recovery after the end of the defense. Trypsin and chymotrypsin are the main enzymes of insect defense in the digestive system, where they are regulated according to the specificity of the PIs in the diet^14,30,61,86^.

Mortality rates were not significantly different, although rTcTI caused high mortality in the second larval instar (45%). Mehmood et al.^87^ also used Kunitz trypsin inhibitors (AnTI) to inhibit the mycelial growth of *Aspergillus niger* and *Fusarium oxysporum*, and found statistically significant mortality rates. This was due the use of a higher concentration of the inhibitor (50 µg) in comparison with that used in this study. Therefore, we can confirm that at concentration of 1.5 µg, rTcTI did not affect the average lifespan of *H. armigera* larvae when fed with leaves containing protease inhibitors, although it caused the death of the larvae in the second instar. This fact may be associated with the concentration of the inhibitor and the incubation time, as well as an exposure response of the larvae to the inhibitor. According to Philippe et al.^74^, insects can develop defense mechanisms and produce proteases that are less susceptible to the action of inhibitors. Other studies have revealed that the tools to control larval growth inhibition do not drastically affect insect mortality. For this reason, the use of these substances can be considered an important strategy for pest control, since they do not interfere in the selection of resistant populations^7,88^.

Spraying protease inhibitors on leaves seems to be an efficient feeding technique. The protease inhibitors are anti-digestive and reduce gut protease activity, thus eliciting compensatory consumption by insects^89^. However, there was no significant difference between the consumed areas of the leaves sprayed with the rTcTI inhibitor compared to the control leaves.

## Conclusion

The present study confirms that TcTI is a trypsin inhibitor of the Kunitz type with excellent potential for biotechnological application due to its biochemical characteristics, such as inhibition of the competitive types, stability at elevated temperatures and high inhibitory capacity.

Its inhibitory effect on the *Mp* fungus indicates its functioning as a defense molecule in the pathosystem *M. perniciosa* × *T. cacao*, in addition to corroborating other studies that have reported its important role in inhibiting the mycelial growth of pathogenic fungi. Studies of its conformation found a secondary structure rich in non-ordered regions, which may be related to the flexibility of the protein in presenting thermostability. These characteristics can be used in the genetic improvement of plants such as *Theobroma cacao*, since it plays an important role as a natural plant defense agent.

## Supplementary Information

Supplementary Figure 1.

Supplementary Figure 2.

Supplementary Figure 3.

Supplementary Figure 4.

Supplementary Table 1.

Supplementary Table 2.

Supplementary Table 3.

## Data Availability

The manuscript has data included as electronic supplementary material.

## Abbreviations

Ki: Inhibition constant
Pi: Protease inhibitors
Mp: *Moniliophthora perniciosa*
