# Supplementary material for: TcTI, a Kunitz-type trypsin inhibitor from cocoa associated with defense against pathogens
Source: Sci Rep. 2022 Jan 13;12:698. doi: 10.1038/s41598-021-04700-y (PMC8758671; doi:10.1038/s41598-021-04700-y)
Supplement: Supplementary file 1 — Supplementary Figure 1. [file 41598_2021_4700_MOESM1_ESM.docx]

**Supplementary figure 1 –** Ramachandran analysis for the pdb structure of TcTI.


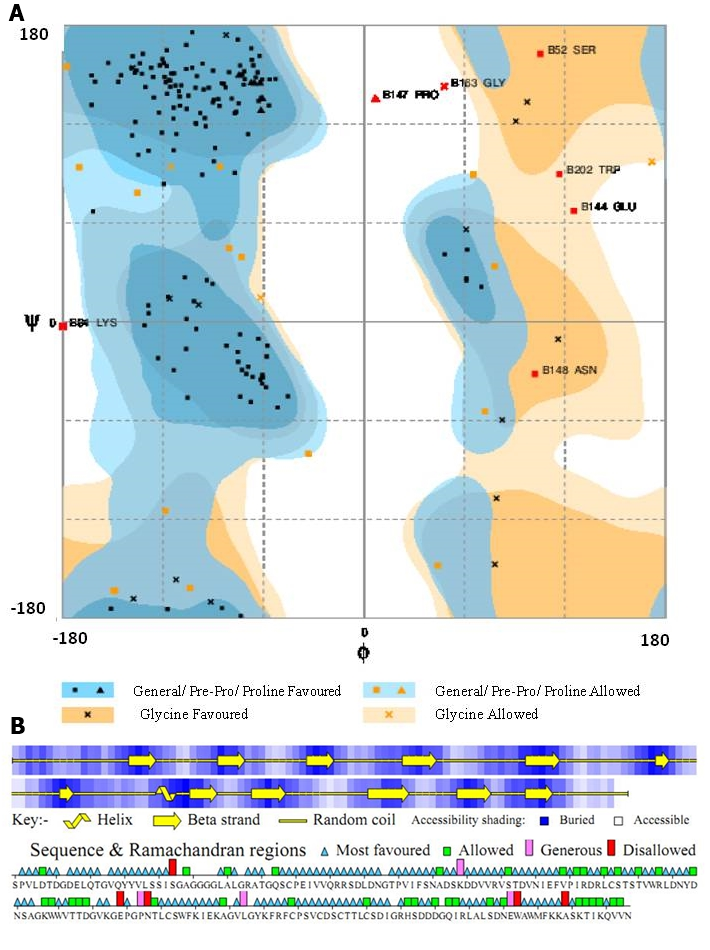


Supplementary data 1: Ramachandran analysis for the pdb structure of TcTI. A- shows the Ramachandran graph, indicating the distribution of the residues in favorable and unfavorable regions. B- shows the most favorable, permissible, possible and unfavorable secondary structure relationships and residue analysis indicated in the figure.
