# Supplementary material for: TcTI, a Kunitz-type trypsin inhibitor from cocoa associated with defense against pathogens
Source: Sci Rep. 2022 Jan 13;12:698. doi: 10.1038/s41598-021-04700-y (PMC8758671; doi:10.1038/s41598-021-04700-y)
Supplement: Supplementary file 2 — Supplementary Figure 2. [file 41598_2021_4700_MOESM2_ESM.docx]

**Supplementary figure 2** – Detection of rare codons for *E.coli* in the EST sequence of TcTI using the ATGme software (<http://atgme.org/>). The rare codons highlighted in orange and very rare codons in red.
