# Supplementary material for: TcTI, a Kunitz-type trypsin inhibitor from cocoa associated with defense against pathogens
Source: Sci Rep. 2022 Jan 13;12:698. doi: 10.1038/s41598-021-04700-y (PMC8758671; doi:10.1038/s41598-021-04700-y)

Supplementary figure 3: 2D gels of the protein profile of different stages of Mp infection in resistant and susceptible cocoa meristems. **a**: Catongo 72 HAI non-inoculated [mock inoculated control]. **b**: Catongo 72 HAI. **c:** TSH1188 72 HAI non-inoculated [mock inoculated control]. **d**: TSH1188 72 HAI. **e**: Catongo 45 DAI non-inoculated [mock inoculated control]. **f**: Catongo 45 DAI. **g**: TSH1188 45 DAI non-inoculated [mock inoculated control]. **h**: TSH1188 45 DAI.

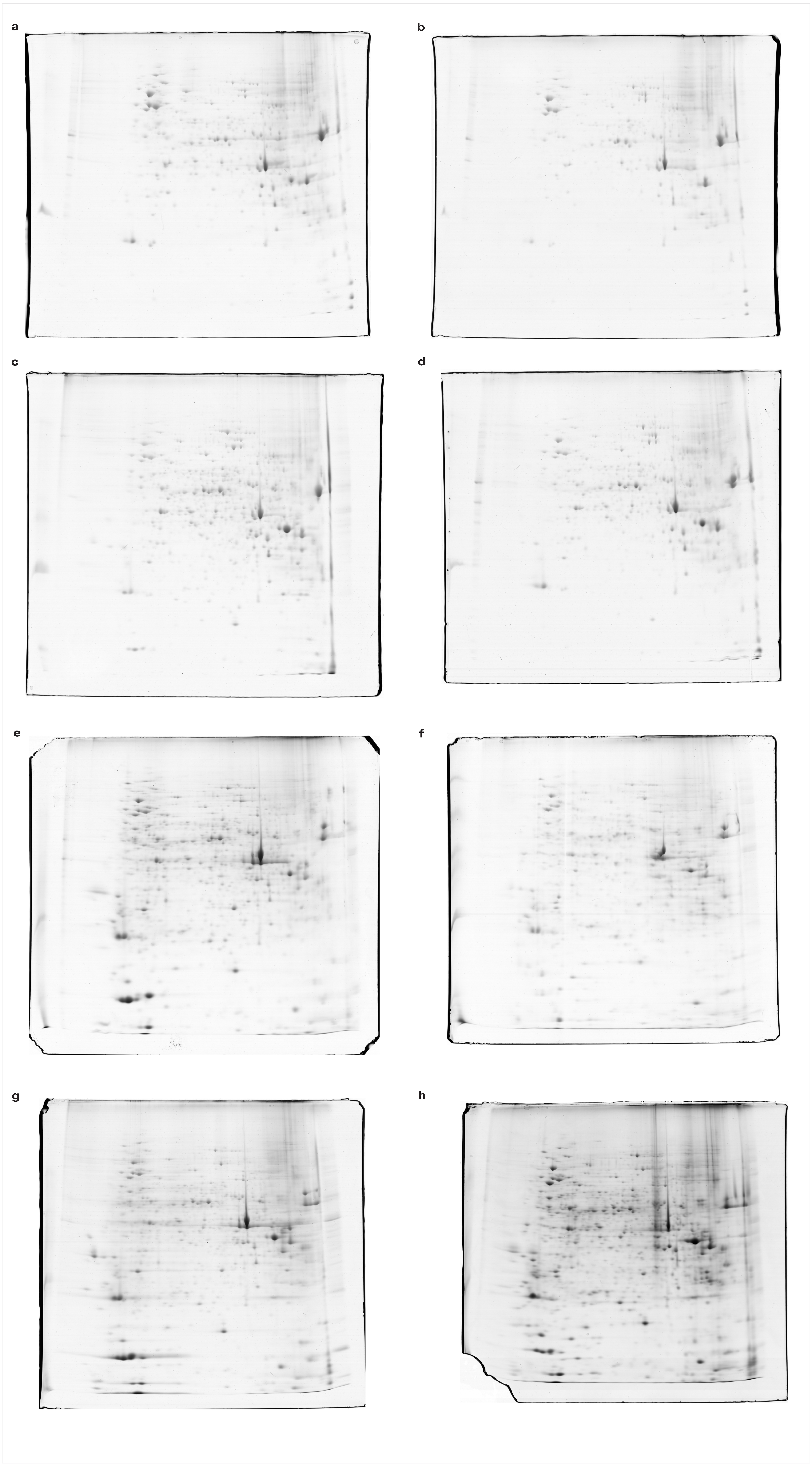

Supplement: Supplementary file 3 — Supplementary Figure 3. [file 41598_2021_4700_MOESM3_ESM.pdf]
