# Supplementary material for: TcTI, a Kunitz-type trypsin inhibitor from cocoa associated with defense against pathogens
Source: Sci Rep. 2022 Jan 13;12:698. doi: 10.1038/s41598-021-04700-y (PMC8758671; doi:10.1038/s41598-021-04700-y)

Supplementary figure 4: Immunodetection of trypsin inhibitors in cocoa meristem of the TSH1188 and Catongo varieties. **a**: Initial stages of 1 and 5 DAI (days after infection); **b**: Final Stages 45 and 60 DAI indicate the periods of infection with *M. perniciosa* and its controls. CAT; Catongo (susceptible variety), TSH; TSH1188 (resistant variety).

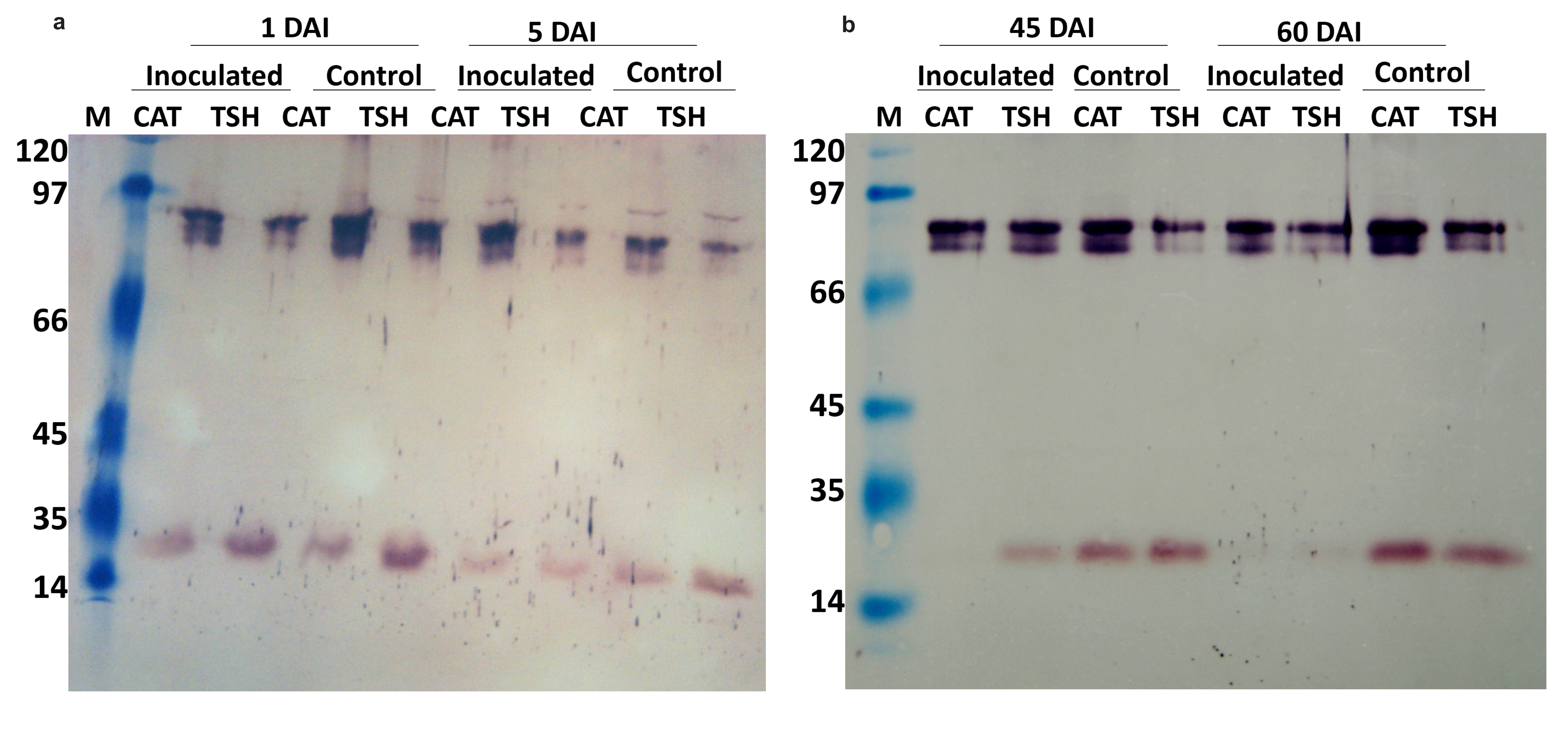

Supplement: Supplementary file 4 — Supplementary Figure 4. [file 41598_2021_4700_MOESM4_ESM.pdf]
