# Supplementary material for: TcTI, a Kunitz-type trypsin inhibitor from cocoa associated with defense against pathogens
Source: Sci Rep. 2022 Jan 13;12:698. doi: 10.1038/s41598-021-04700-y (PMC8758671; doi:10.1038/s41598-021-04700-y)
Supplement: Supplementary file 7 — Supplementary Table 3. [file 41598_2021_4700_MOESM7_ESM.docx]

**Supplementary table 3**: Larval instar date. Weight (g), leaf area after consumption (cm^2^) and days from hatch to larva death.

|  |  | **Weight g** | | | | **Area cm^2^** | **Hatch-to-death duration (days)** |
| --- | --- | --- | --- | --- | --- | --- | --- |
| **Treatment** | **Biological repetition** | **Experiment setup day** | **24h later** | **3 days later** | **7 days later** | **Leaf consumption** | **Mortality** |
| Control | C1 | 0,00234 | 0,00288 | 0,00353 | 0,01236 | 2,85 | 25 |
| Control | C2 | 0,00142 | 0,00205 | 0,00203 | 0,0205 | 2,04 | 13 |
| Control | C3 | 0,00351 | 0,00489 | 0,01757 | 0,10204 | 6,12 | 14 |
| Control | C4 | 0,00413 | 0,0051 | 0,01689 | 0,12083 | 2,22 | 24 |
| Control | C5 | 0,0028 | 0,0036 | 0,00837 | 0,02146 | 2,03 | 21 |
| Control | C6 | 0,00808 | 0,00826 | 0,00688 | 0,39605 | 3,53 | 20 |
| Control | C7 | 0,00177 | 0,00111 | 0* | 0* | 3,78 | 26 |
| Control | C8 | 0,00377 | 0,00407 | 0* | 0* | 0,52 | 0* |
| Control | C9 | 0,01175 | 0,01942 | 0,05194 |  | 2,44 | 22 |
| Control | C10 | 0,01347 | 0,01222 | 0,01625 | 0,02872 | 2,24 | 18 |
| Control | C11 | 0,00131 | 0,00144 | 0,00134 | 0* | 6,95 | 22 |
| Control | C12 | 0,0017 | 0,0028 | 0,00246 | 0,42077 | 2,12 | 21 |
| Control | C13 | 0,00099 | 0,00177 | 0,0066 | 0,08057 | 0* | 8 |
| TcTI cacao | C1 | 0,0076 | 0,00887 | 0,02113 | 0,11533 | 0,54 | 24 |
| TcTI cacao | C2 | 0,00949 | 0,01232 | 0,03235 | 0* | 3,27 | 16 |
| TcTI cacao | C3 | 0,00491 | 0,01642 | 0,04966 | 0* | 1,74 | 22 |
| TcTI cacao | C4 | 0,00495 | 0,00705 | 0,00607 | 0,00689 | 5,19 | 22 |
| TcTI cacao | C5 | 0,00891 | 0,01249 | 0,03827 | 0,19075 | 1,41 | 22 |
| TcTI cacao | C6 | 0,00904 | 0,01233 | 0,04052 | 0,26142 | 1,72 | 13 |
| TcTI cacao | C7 | 0,00471 | 0,00619 | 0,0055 | 0,00829 | 0,61 | 8 |
| TcTI cacao | C8 | 0,00066 | 0,00066 | 0* | 0* | 0* | 8 |
| TcTI cacao | C9 | 0,00185 | 0,00301 | 0,00281 | 0,00294 | 3,41 | 22 |
| TcTI cacao | C10 | 0,000204 | 0,00363 | 0,01116 | 0,16768 | 2,42 | 0* |
| TcTI cacao | C11 | 0,01249 | 0,02613 | 0,07316 | 0,46059 | 2,61 | 13 |
| TcTI cacao | C12 | 0,0026 | 0,00389 | 0,01238 | 0,1457 | 1,5 | 23 |
| TcTI cacao | C13 | 0,0058 | 0,00506 | 0* | 0* | 2,23 | 22 |

* Larva death
